# Genomic landscape, immune characteristics and prognostic mutation signature of cervical cancer in China

**DOI:** 10.1186/s12920-022-01376-9

**Published:** 2022-11-04

**Authors:** Jing Liu, Zirong Li, Ting Lu, Junping Pan, Li Li, Yanwen Song, Dan Hu, Yanhong Zhuo, Ying Chen, Qin Xu

**Affiliations:** 1 Department of Gynecology, Clinical Oncology School of Fujian Medical University, Fujian Cancer Hospital, No. 420 Fuma Road, Jinan District, Fuzhou, 350014 China; 2 Department of Radiation Oncology, Longyan First Hospital, Affiliated to Fujian Medical University, Longyan, Fujian China; 3 Department of Pathology, Fujian Women and Children Hospital, Fuzhou, Fujian China; 4 Department of Radiation Oncology, Clinical Oncology School of Fujian Medical University, Fujian Cancer Hospital, No. 420 Fuma Road, Jinan District, Fuzhou, 350014 China; 5 Department of Pathology, Clinical Oncology School of Fujian Medical University, Fujian Cancer Hospital, No. 420 Fuma Road, Jinan District, Fuzhou, 350014 China; 6 Department of Radiotherapy, Zhangzhou Hospital, Teaching Hospital of Fujian Medical University, Zhangzhou, 363000 Fujian China

**Keywords:** Cervical cancer, Targeted therapies, PD-L1 expression, Gene mutation, Tumour mutation burden

## Abstract

**Purpose:**

This study aimed to analyse the genomic alteration profiles and immune characteristics of a cohort of Chinese cervical cancer patients to understand why certain patients benefited from molecular targeted therapies and immunotherapy as well as their prognostic significance.

**Methods:**

PD-L1 expression and clinicopathological information were obtained from 98 cervical cancer patients. Differences in PD-L1 expression and gene mutations between squamous cell carcinoma (SCC) and adenocarcinoma (AC) were analysed by the chi-square test or Fisher's exact test. Differences in gene mutations between our cohort and The Cancer Genome Atlas (TCGA) cohort were tested by Fisher's exact test. Logistic regression was used to analyse factors influencing TMB-high.

**Results:**

Positive PD-L1 expression was significantly higher in cervical SCC than in cervical AC (87% vs. 39%, *p* < 0.001). Frequently mutated genes in cervical cancer included the *PIK3CA, KMT2D*, and *KMT2C* genes, among others. *PIK3CA* gene mutation rates were significantly higher in SCC than in AC (*p* = 0.004). The *TERT* gene mutation rate was significantly higher in our cohort than in the TCGA cohort (12% vs. 1%, *p* < 0.001). The independent predictors of high TMB were *KMT2C* and *LRP1B* gene mutations (*p* < 0.05). We also found that PTEN mutations were associated with worse survival (median PFS, 12.16 vs. 21.75 months, *p* = 0.0024).

**Conclusion:**

Cervical SCC and AC have different molecular profiles and immune characteristics, suggesting that targeted treatments for SCC and AC patients may improve clinical outcomes. *KMT2C* and *LRP1B* gene mutations are independent predictors of TMB-high status in cervical cancer. We also proposed the prognostic value of PTEN mutations.

**Supplementary Information:**

The online version contains supplementary material available at 10.1186/s12920-022-01376-9.

## Background

Cervical cancer is the fourth most common cancer and has a high mortality rate among women worldwide. In China, cervical carcinoma is the second most common malignancy among women [1, 2]. Early cervical cancer can be effectively treated by surgery, while concurrent chemoradiation is the first choice for locally advanced cervical cancer. However, 20–30% of cervical cancer patients experience recurrence or distant metastasis after first-line treatment, and the treatment strategies for these patients are limited. Therefore, local recurrence and distant metastasis are the major causes of death in advanced cervical carcinoma.

In recent years, molecularly targeted therapies have been increasingly applied to various malignancies. The comprehensive genetic and molecular characteristics of malignant cervical tumours were described by The Cancer Genome Atlas Research Network [3]. Nevertheless, there were only 20 Asians in the TCGA cohort, and the TCGA data only published the sequencing results, without analysing the differences between adenocarcinoma and squamous cell carcinoma. In China, only a few studies with small sample sizes or sequencing of a small panel of genes have evaluated gene mutations in cervical cancer. Therefore, it is necessary to establish a larger genetic profile of the Chinese population through next-generation sequencing (NGS).

Immunotherapy is an emerging treatment for cervical cancer. However, the KEYNOTE-158 trial showed that the overall response rate of cervical cancer patients treated with immunotherapy was only 12.2% [4]. Malignant tumour patients with positive PD-L1 expression and high tumour mutational burden (TMB) were considered a patient population that may achieve a sustained benefit from pembrolizumab [5]. Therefore, it is critical to distinguish cervical cancer patients who are more likely to benefit from immune checkpoint inhibitors. However, only a few studies with small samples have reported PD-L1 expression and TMB in Chinese populations with cervical cancer [6, 7].

Squamous cell carcinoma (SCC) and adenocarcinoma (AC) are the prime pathological classifications of cervical carcinoma. Some studies have shown that AC has higher rates of lymph node involvement and distant metastasis than SCC, and the prognosis of AC is poor [8–10]. It was reported in a study subgroup analysis that SCC patients have a better response to immunotherapy than AC patients [11]. Only a few studies have investigated whether SCC and AC have different molecular profiles among Chinese patients.

The incidence and spectrum of somatic mutations in carcinoma of the cervix were analysed in the current study. We compared the differences in the molecular profiles, PD-L1 expression, and TMB between cervical SCC and AC in a Chinese population. These findings may help guide the targeted therapy of cervical cancer and lead to the identification of new markers that can predict the therapeutic effect of immune checkpoint inhibitors in cervical cancer patients by integrating the expression of PD-L1, genomic variation and TMB.

## Methods

### Samples and clinical data

We enrolled 98 patients with cervical cancer in Fujian Cancer Hospital. Clinical data were extracted from electronic medical records. Samples including tumour tissues as well as matched neighbouring normal tissues or peripheral blood were collected.

### Immunohistochemistry

Immunohistochemical analysis of PD-L1 expression was performed on 4–5 μm formalin-fixed and paraffin-embedded sections using an anti-human PD-L1 antibody (Dako 22C3) according to the manufacturer’s recommendations. The combined positive score (CPS) was used to measure PD-L1 protein expression. The number of PD-L1-stained cells was divided by the total quantity of surviving tumour cells and then multiplied by 100. Based on previous studies [11], we set two cut-off values of CPS for PD-L1 expression: 1 and 10. PD-L1 positivity was defined as CPS ≥ 1.

### ***Library construction and sequencing ***[12]

A total amount of 1.0 μg genomic DNA per sample was used as input material for the DNA sample preparation. Sequencing libraries were generated using a customized panel that targeted 484 cancer genes. Briefly, fragmentation was carried out by a hydrodynamic shearing system (Covaris, Massachusetts, USA) to generate 180–280 bp fragments. The remaining overhangs were converted into blunt ends via exonuclease/polymerase activities, and enzymes were removed. After adenylation of the 3’ ends of DNA fragments, adapter oligonucleotides were ligated. DNA fragments with ligated adapter molecules on both ends were selectively enriched in a PCR. Captured libraries were enriched in a PCR to add index tags to prepare for hybridization. The products were purified using an AMPure XP system (Beckman Coulter, Beverly, USA) and quantified using an Agilent high-sensitivity DNA assay on an Agilent Bioanalyzer 2100 system. Libraries were sequenced with standard 2 × 150-bp paired-end reads on the Illumina NovaSeq 6000 sequencer. Sequencing was performed with 1000×–2000×  depth on targeted 3 Mb regions.

### Genome alignment and variant calling

After removing sequencing reads with low quality and adapter bases using FASTP (https://github.com/OpenGene/fastp), clean reads were aligned to the human genome (National Center for Biotechnology Information build 37, hg19) using the Burrows–Wheeler Aligner (https://github.com/lh3/bwa). Sorted BAM files were created using sambamba (https://github.com/biod/sambamba). Duplicate reads were marked using samblaster (https://github.com/GregoryFaust/samblaster). Single nucleotide variants (SNVs) and small insertions and deletions (indels) were called and identified using Varscan2 (http://varscan.sourceforge.net). False-positive mutations were removed using in-house filter tools and IGV (http://www.igv.org) double-checked manually with variant allele frequency ≥ 1%.

### Functional annotation

We used ANNOVAR (https://annovar.openbioinformatics.org/en/latest/) to annotate the variant call format. Consensus Coding Sequence and RefSeq were used to determine amino acid variations. The annotation content contained variant positions, variant types, and other information. The dbSNP (http://www.bioinfo.org.cn/relative/dbSNP%20Home%20Page.htm), COSMICV91 (https://cancer.sanger.ac.uk/cosmic), 1000 Genomes Project (https://www.internationalgenome.org), and Exome Aggregation Consortium (http://exac.broadinstitute.org) databases were also used to obtain the population frequencies and other information for the mutations.

### Open-source dataset acquisition and preprocessing

The MAF files and clinical information of cervical cancer patients were obtained from the TCGA database (https://portal.gdc.cancer.gov). To remove the interbatch effect (TCGA uses whole-exome sequencing (WES) with middle depth, while this project used the Novogene PM2.0 panel with high depth), we extracted mutations of pm2.0-covered genes in the TCGA data using Python 2.7 (Additional file 1: Table S1).

### Visualization and data statistics

We used the R package maftools (http://bioconductor.org/packages/release/bioc/vignettes/maftools/inst/doc/maftools.html) for statistical analysis and visualization, including landscape analyses, statistical testing, and other analyses.

### Identification of significantly mutated genes

MuSiC package was performed to identify frequently mutated genes that show a significantly higher mutation rate than the background mutation rate (BMR). BMR was calculated as the mutations per Mb in the coding and splicing regions of 484 genes and also in the categories of AT/CG/CpG transitions, AT/CG/CpG transversions, and Indels. MuSiC applied three SMG tests, which include a convolution test (CT), a Fisher’s combined p value test (FCPT) and a likelihood ratio test (LRT), to summarize *p* values of mutational significance. Genes were considered to be SMGs with FDR < 0.05 in any of the three tests.

### Calculation of TMB

The TMB of cervical cancer patients was calculated as the ratio of the total amount of nonsynonymous mutations to the total coding size of the panel (which was 1.2 Mb for the Novogene PM2.0 483 gene panel). The mutation count included nonsynonymous SNVs and indels detected within the coding region, with the exclusion of driver gene events (in the *EGFR, MET, BRAF, PIK3CA, NF1, KRAS, TP53, NOTCH1, NOTCH2, NOTCH3*, and *NOTCH4* genes). Only mutations with allelic fractions of ≥ 2.5% for SNVs and 1.3% for indels were included in the mutation count. TMB cutoff value was defined based on pan-cancer datasets (191 colorectal cancer cases, 297 lung cancer cases, and 326 other cancer cases) which were sequenced with the same panel. TMB was divided into high or low with the top quartile as the cut-off value (5.19 muts/MB).

### Statistical analysis

Statistical analyses were conducted using GraphPad PRISM (version 8.0.2) and SPSS (version 25.0). Mutation information analysis was performed using R software (version 4.0.1). The differences in PD-L1 expression and gene mutations between SCC and AC were analysed by the chi-square test or Fisher's exact test. The Benjamini–Hochberg method was used to adjust *P* values for all the tests when multiple testing was concerned. Differences in mutations between our cohort and the TCGA cohort were tested by Fisher's exact test. The relationship between gene mutations and TMB was analysed by the Mann–Whitney U test. Factors influencing TMB-high were analysed by logistic regression. Progression-free survival (PFS) curves were plotted by the Kaplan–Meier method, and the difference in survival between the two groups was analysed by the log-rank test. Univariate and multivariate Cox regression analyses were conducted to test whether PTEN mutation was an independent prognostic factor. For all analyses, clinical stages were categorized as stages I-II or III-IV. Histological types were categorized as SCC or non-SCC. Age was a continuous variable. A *p* value < 0.05 was considered significant.

## Results

### Clinical characteristics and pathological data

The clinical characteristics and pathological data of 98 patients are summarized in Additional file 2: Table S2. The median age was 50.5 years (range from 34 to 73). Of 98 patients, 67 patients (68.4%) had squamous cell carcinoma (SCC), 26 patients (26.5%) had adenocarcinoma (AC), and 5 patients (5.1%) had adenosquamous carcinoma (ASC). At initial diagnosis, there were 16 (16.3%) patients in stage I, 34 (34.7%) in stage II, 31 (31.6%) in stage III, and 11 (11.2%) in stage IV. PD-L1 positivity was found in 54 (72.0%) of 75 patients. The PD-L1 expression status was unknown in 23 patients.

### Landscapes of frequently mutated genes in our cohort

**Fig. 1:**
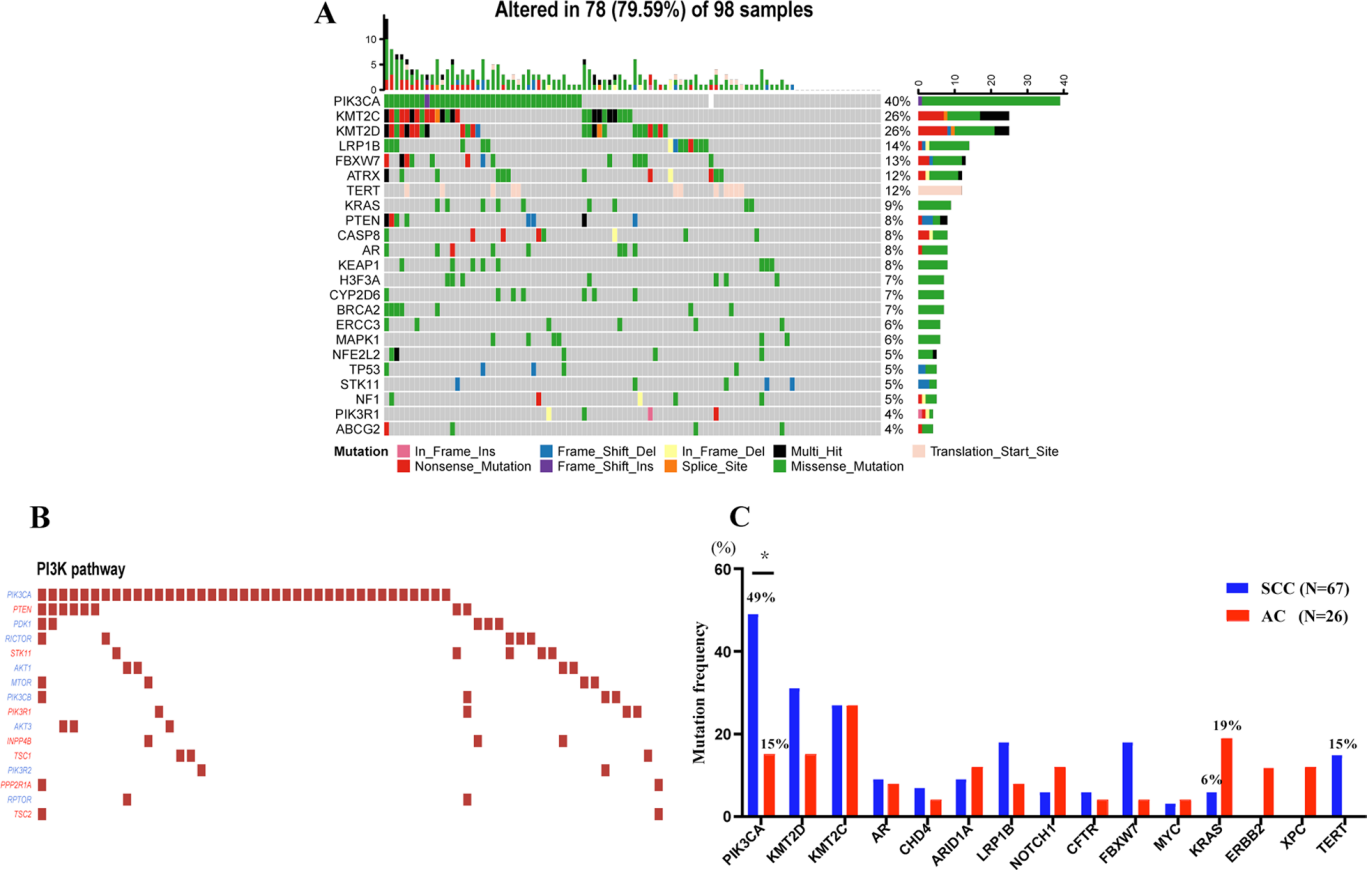
Landscapes of frequently mutated genes in cervical cancer. **A** Oncoplot displaying the landscapes of frequently mutated genes. Genes are ordered according to the mutation frequency (left panel), and different mutation types are indicated by the annotation bar (bottom). **B** Genomic mutations of the PI3K/mTOR genes in cervical cancer. **C** Differences in mutations between adenocarcinoma (AC) and squamous cell carcinoma (SCC). ^*^p < 0.05

We performed mutation spectrum analysis in 98 cervical cancer samples, including 26 AC samples, 67 SCC samples, and 5 ASC samples. Validated gene mutations were detected in 91 of the 98 tumours (93%). The top 20 most frequently mutated genes are shown in Fig. 1A. The five genes with the highest mutation frequency in the overall samples were the *PIK3CA* (40%), *KMT2D* (26%), *KMT2C* (26%), *LRP1B* (14%), and *FBXW7* (13%) genes. The gene mutations were mostly present in genes involved in the PI3K-AKT signalling pathway. Activation of *PIK3CA* mutations in cancer predominantly leads to the dysregulation of the PI3K/AKT/mTOR pathway (Fig. 1B).

### Differences in gene mutations between AC and SCC in our cohort

The distributions of gene mutations detected in AC and SCC are shown in Fig. 1C. We observed a higher frequency of PIK3CA gene mutation in SCC than in AC (49% vs. 15%, *p* = 0.004, adjust *p* value = 0.049). We also detected a trend towards more patients with SCC harbouring *TERT* gene mutations (15% vs. 0%, *p* = 0.057, adjust *p* value = 0.512), Although the difference was insignificant after multiple test adjustments.

### Gene mutation differences between our cohort and the TCGA cohort

**Fig. 2:**
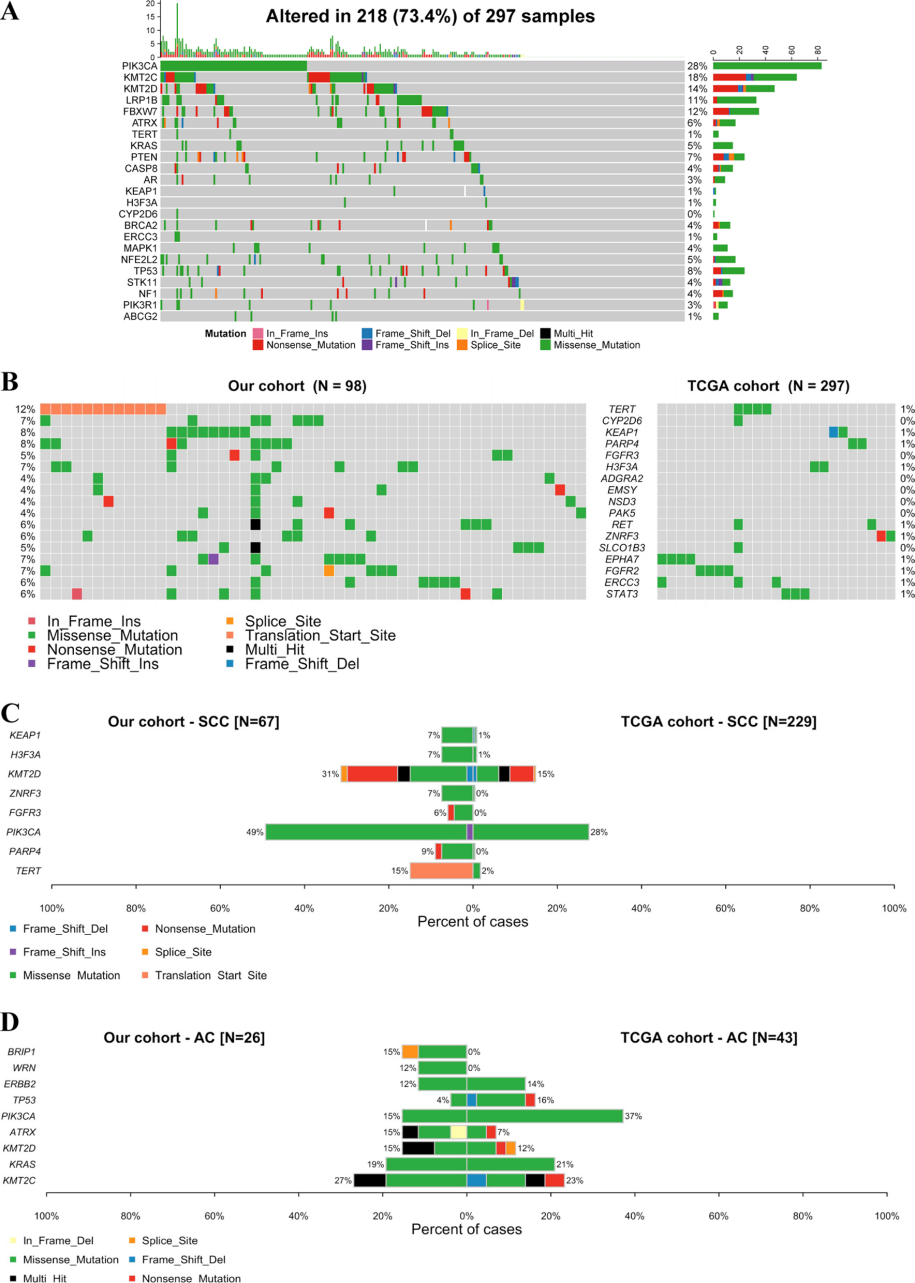
Landscapes of frequently mutated genes in cervical cancer from TCGA and differences in mutations between our cohort and the TCGA cohort. **A** Oncoplot displaying the landscapes of frequently mutated genes from the TCGA cohort. Genes are ordered according to the mutation frequency (left panel), and different mutation types are indicated by the annotation bar (bottom). **B** Gene mutation differences between our cohort and the TCGA cohort. Comparison of gene mutations between our cohort and the TCGA cohort in cervical squamous cell carcinoma (SCC) (**C**) and cervical adenocarcinoma (AC) (**D**)

The top 20 most frequently mutated genes in the TCGA cohort are shown in Fig. 2A. We observed a relatively consistent pattern of the frequently mutated genes in the TCGA cohort compared with our cohort. Notably, a higher *TERT* gene mutation frequency was detected in our cohort than in the TCGA cohort (12% vs. 1%, *p* < 0.001), especially in SCC (15% vs. 2%, *p* < 0.001) (Fig. 2B, C).

We also compared gene mutations among the different histological subtypes between our cohort and the TCGA cohort. There has a different histological distribution between the TCGA cohort and our cohort. In our cohort, 67 patients (68.4%) had SCC, 26 patients (26.5%) had AC. In TCGA, 229 patients (77.1%) had SCC, 43 patients (14.5%) had AC. In cervical SCC, the frequencies of *PIK3CA*, *KMT2D* and *FGFR3* gene mutations were significantly higher in our cohort than in the TCGA cohort (all *p* < 0.05) (Fig. 2C). In cervical AC, significantly higher frequencies of *BRIP1* and *WRN* gene mutations, which are targets of PARP inhibitors, were detected in our cohort than in the TCGA cohort (15% vs. 0%, *p* = 0.017; 12% vs. 0%, *p* = 0.049, respectively). In AC, we observed a lower *PIK3CA* mutation frequency in our cohort than in the TCGA cohort (15% vs. 37%, *p* = 0.061). The rates of *TP53* and *ERBB2* mutations in our cohort were consistent with those in the TCGA cohort (Fig. 2D).

### Differences in PD-L1 expression between cervical SCC and AC

**Fig. 3:**
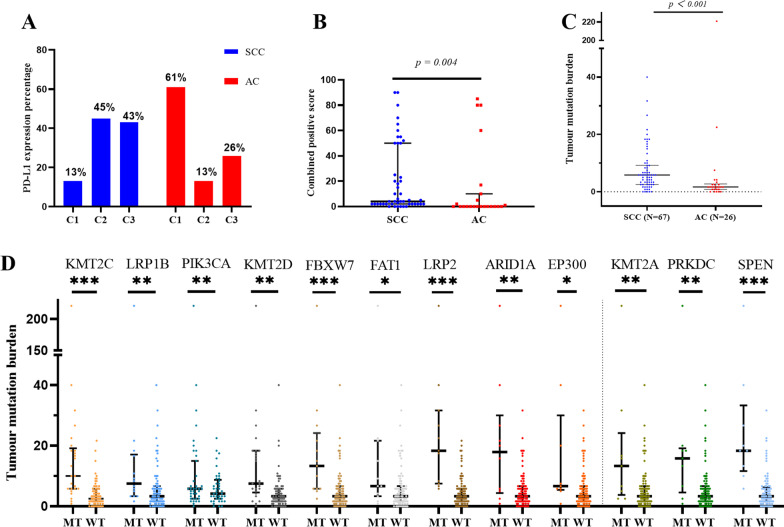
PD-L1 expression and tumour mutational burden (TMB) in cervical cancer. **A** Distribution of PD-L1 expression in different histological subtypes of cervical cancer. Comparison of PD-L1 expression (**B**) and TMB (**C**) between cervical squamous cell carcinoma (SCC) and cervical adenocarcinoma (AC). C1: CPS < 1; C2: 1 ≤ CPS < 10; and C3: CPS ≥ 10. (D) Associations between TMB and mutations in the *KMT2C*, *LRP1B, PIK3CA, KMT2D, FBXW7, FAT1, LRP2, ARID1A, EP300, KMT2A, PRKDC*, and *SPEN* genes. *p < 0.05; **p < 0.01; ***p < 0.001. WT, wild-type; MT, mutant type

Among the 98 cervical cancer samples, 75 patients were tested for PD-L1 expression, and the rate of positive PD-L1 expression was 72% (54/75) (Additional file 2: Table S2). Specimens were stratified into the following three groups (C1–3) based on the cut-off values for PD-L1 expression as defined in the Methods section: C1: CPS < 1; C2: 1 ≤ CPS < 10; and C3: CPS ≥ 10. The distribution of cervical cancer samples in each group was as follows: 28% (21/75) in C1; 35% (26/75) in C2; and 37% (28/75) in C3. The expression of PD-L1 in cervical AC and cervical SCC in the different subgroups is shown in Fig. 3A. The rate of positive PD-L1 expression in cervical SCC was significantly higher than that in cervical AC (87% vs. 39%, *p* < 0.001), and the expression intensity of PD-L1 in cervical SCC was significantly higher than that in cervical AC (median CPS: 4 vs. 0, *p* = 0.004) (Fig. 3B).

### Differences in TMB between cervical SCC and AC

We defined TMB-high (TMB-H) as the top 25% of the TMB value of the samples in pan-cancer datasets. Among the 98 cervical cancer samples, 40% (39/98) were TMB-H, and 60% (59/98) were TMB-low. In comparing the difference in TMB between cervical SCC and AC, we found that SCC showed a significantly higher TMB than AC (median TMB: 5.830 vs. 1.670, *p* < 0.001) (Fig. 3C).

### KMT2C and LRP1B gene mutations are associated with increased TMB in cervical cancer

We next explored the correlation between gene mutations and TMB in cervical cancer by analysing the top 15 most frequently mutated genes. *KMT2C* gene mutation in cervical cancer was associated with higher TMB levels than in cervical cancers with wild-type *KMT2C* (median 10 vs. 2.5,* p* < 0.001). Similar results were observed with *LRP1B, PIK3CA, KMT2D, FBXW7, FAT1, LRP2, ARID1A, KMT2A, PRKDC, SPEN*, and *EP300* gene mutations (all *p* < 0.05) (Fig. 3D).

To validate the association of gene mutations with TMB values, logistic regression was performed to analyse the factors that influence TMB-H. We analysed the impact of histological subtype by dividing subtypes into SCC and non-SCC. The results indicated that the independent risk factors for TMB-H were KMT2C gene mutation and LRP1B gene mutation (all *p* < 0.05) (Table 1).

**Table 1:** Univariate and multivariate logistic regression of factors influencing TMB-high in cervical cancer

Factor	Univariate		Multivariate	
	OR (95%CI)	*p* value	OR (95%CI)	*p *value
Histological subtypes (scc, non-scc)	5.358(1.837–15.623)	0.002	4.767(0.992–22.902)	0.051
KMT2C (mutant, wild)	11.368(3.744–34.520)	< 0.001	13.160(2.814–61.553)	0.001
LRP1B (mutant, wild)	3.240(0.995–10.553)	0.051	5.981(1.311–27.291)	0.021
PIK3CA (mutant, wild)	2.657(1.149–6.148)	0.022	1.208(0.359–4.062)	0.760
FBXW7 (mutant, wild)	11.196(2.322–53.983)	0.003	5.338(0.819–34.764)	0.080
PRKDC (mutant, wild)	6.234(1.221–31.820)	0.028	1.966(0.240–16.122)	0.529
KMT2D (mutant, wild)	6.367(2.320–17.473)	< 0.001	2.520(0.663–9.574)	0.175
EP300(mutant, wild)	6.234(1.221–31.820)	0.028	7.876(0.997–62.241)	0.050

*Non-scc* Non squamous cell carcinoma, *scc* Squamous cell carcinoma

### Correlations of PTEN mutations and PD-L1 expression with clinical outcomes

**Fig. 4:**
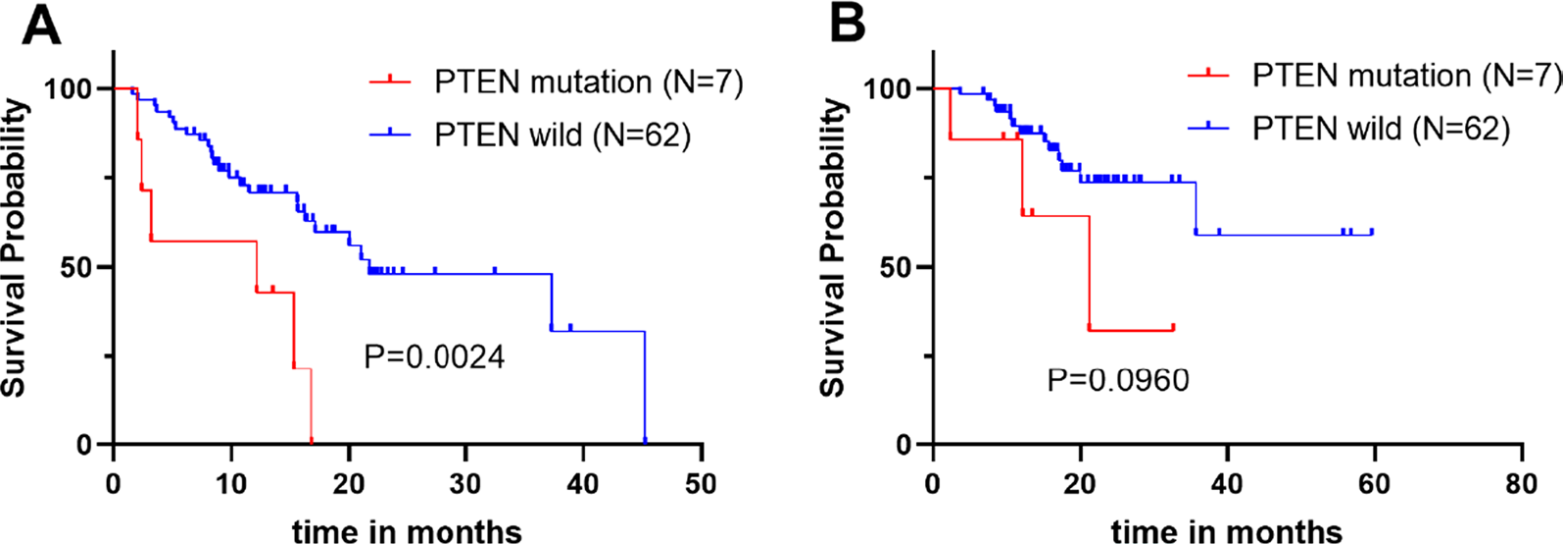
Kaplan–Meier estimated **A** PFS and **B** OS by PTEN mutational status

We next analysed the correlation between genomic mutations and clinical outcomes. A total of 29 patients were excluded because gene mutations were not detected in 7 patients, 9 patients were lost to follow-up, and 13 patients received immunotherapy treatment. In 69 patients, PTEN mutations (7/69, 10.1%) were associated with poorer PFS (12.16 vs. 21.75 months, log-rank test *p* = 0.0024) and tended to worsen OS (log-rank test *p* = 0.096) compared to wild-type PTEN (Fig. 4A, B). To construct the Cox proportional hazards model, previously reported prognostic factors, such as age (continuous value), histological type (non-SCC vs. SCC), and stage, were used as adjustment factors during the analysis. In multivariate analysis, PTEN mutations were correlated with shorter PFS (hazard ratio 3.946, 95% CI, 1.500–10.380, *p* = 0.050) (Table 2). This suggests that PTEN mutations are an independent predictive factor of poorer clinical outcome in our patients.

**Table 2:** Univariate and multivariate analyses of clinical parameters on progression-free survival (PFS) (Cox regression) (N = 69)

Variable	Univariate		Multivariate	
	HR (95% CI)	*p value*	HR (95% CI)	*p value*
Age	0.999(0.956–1.044)	0.955	0.979(0.934–1.026)	0.374
Histological types (non-SCC vs SCC)	2.031(0.771–5.353)	0.1433	1.702(0.621–4.660)	0.301
Stage (III-IV vs I-II)	1.864(0.905–3.842)	0.091	1.679(0.794–3.550)	0.175
PTEN (mutant, wild)	3.765(1.501–9.446)	0.005	3.946(1.500–10.380)	0.005

A survival analysis was also performed in 54 patients by PD-L1 expression status. A total of 21 patients were excluded because 8 patients were lost to follow-up and 13 patients received immunotherapy treatment. There was no significant difference between PD-L1-positive and PD-L1-negative patients in terms of OS (log-rank test *p* = 0.1684). However, shorter PFS was observed in patients with positive PD-L1 expression (log-rank test *p* = 0.0267) (Additional file 3: Fig. S1).

## Discussion

There are limited data on the genomic alteration profiles of cervical cancer in Chinese patients. In this study, cervical cancer samples from 98 Chinese patients were analysed using NGS and immunohistochemistry to identify the characteristic features of gene alterations as well as TMB and PD-L1 expression. Significant differences in the personal genomic landscapes were detected across cervical cancer subtypes, and our results indicated that mutations in the *KMT2C* and *LRP1B* genes were associated with higher TMB values. In addition, our findings suggest that PTEN mutations and PD-L1 expression are related to the prognosis of patients.

PD-L1 expression determined by immunohistochemistry is a validated biomarker with a strong correlation to the response to immune checkpoint inhibitors. A previous report showed that the proportion of positive PD-L1 expression (≥ 50%) in Chinese patients with non-small-cell lung cancer might be different from that in patients from Western countries [13]. In KEYNOTE 158, the overall response rate of cervical cancer patients with PD-L1-positive tumours (CPS ≥ 1) to immunotherapy was 14.6% [4]. Previous studies have indicated that the efficacy of immune checkpoint inhibitor therapies is limited in cervical cancer patients [14]. According to previous reports, PD-L1 was expressed in 34.4–96% of cervical cancer tissues, while it was rarely found in normal cervical tissues [15]. Helen and other researchers reported PD-L1 positivity in 83 of 154 (54%) SCC patients and 7 of 49 (14%) AC patients (*p* < 0.001) [16]. Consistent with these data, we also noticed that PD-L1 expression was more frequent in SCC (87%) than in AC (39%), and the median CPS value in SCC (4) was higher than that in AC (0). Similar to the findings of previous studies, the results of our study showed that patients who were PD-L1-negative had a better prognosis [17]. Nonetheless, the results of our prospective clinical study suggested that patients with high PD-L1 expression had a better ORR (70.4% vs. 33.3%; *P* = 0.041) and longer PFS (*P* = 0.014) than those with low expression in antivascular therapy combined with anti-PD-1 therapy (unpublished observations).^1^

Importantly, our study showed that the spectrum of genomic alterations in Chinese patients with cervical cancer was roughly the same as that in patients from Western countries. In our study, the *PIK3CA, KMT2C, KMT2D, LRP1B*, and *FBXW7* genes were the five genes with the highest mutation frequencies in cervical cancer samples, which is mostly consistent with the results in TCGA. However, we found a substantially higher TERT alteration frequency in our study than in TCGA. One reason for this result may be that the TERT mutations occurred in the promoter, and while WES is unable to detect mutations in the promoter, we were able to detect these mutations using NGS. To the best of our knowledge, this is the first study to report a TERT promoter mutation in a Chinese population with cervical cancer. By WGS data, TERT hotspot mutations were found in 74% of primary glioblastoma cases in a previous study, and these mutations resulted in increased TERT RNA expression [18, 19]. The same hotspot mutation was also found in our research by NGS. These results indicate that TERT mutations may represent a therapeutic target for cervical cancer.

We also compared the frequency of mutations among histological subgroups of cervical cancer in this study. The prognosis of AC is worse, which is probably because of the differences in genetic mutations between subgroups. Our results revealed different characteristics of gene mutations in different pathological types of cervical cancer. *PIK3CA* gene alterations were considerably higher in SCC (49%) than in AC (15%). The secondary analysis of CLAP trial also suggested that same result [20]. *PIK3CA* mutations occurred most frequently in the overall patient group, and the mutation rate in our study was higher than the rates in previous studies of cervical carcinoma from the Netherlands (20%), France (27%), Latin America (28–33%), the USA (31%) and Norway (15%) [3, 21–24]. These differences may be related to differences in race, ethnicity, geography, tumour characteristics, or staging. *PIK3CA* is not only one of the PI3K/AKT/mTOR pathway members but is also the most frequently mutated oncogene in human cancers [25]. In vitro studies have shown that the activated PI3K/AKT/COX-2 pathway may induce human AC HeLa cells to develop resistance to radiation [26]. Nusrat and other researchers recently reported that microsatellite stable colorectal cancer patients with *PIK3CA* mutations benefited from immunotherapy [27]. Another study showed that PIK3CA mutations were associated with the response to immunotherapy in cervical cancer [7]. However, only 32 patients were included in this analysis. Therefore, the relationship between *PIK3CA* mutations and immunotherapy needs to be further investigated in a study with a larger sample size. Our results also showed that the frequency of TERT gene mutations was significantly higher in SCC than AC.

We also reported for the first time the difference in the frequency of *FGFR3* mutations between cervical SCC patients in our cohort and the TCGA database cohort. Patients with *FGFR3* mutation may have a relatively worse prognosis after chemotherapy and immune checkpoint inhibition [28]. A recent clinical trial of the selective FGFR1-3 inhibitor infigratinib for the treatment of FGFR3-altered metastatic breast cancer as second-line therapy showed that the overall response rate was as high as 25% [29]. FGFR3 inhibitors may thus represent a potential therapeutic method for cervical cancer patients with *FGFR3* mutations. The mutation frequency of the *KMT2D* gene was significantly higher in our group than in TCGA. Wang et al. suggested that *KMT2D* deficiency makes tumours more sensitive to immune checkpoint blockade by enhancing tumour immunogenicity [30]. Our data indicated that KMT2D mutation was also related to higher TMB in cervical cancer. Moreover, the results of a prospective study in our centre suggested that patients with KMT2D mutations had a better ORR (*p* < 0.05) with antivascular therapy combined with anti-PD-1 therapy.^2^ These results could help identify a sizeable patient subpopulation that may show sensitivity to immune checkpoint inhibition.

We also found significantly higher *BRIP1* and *WRN* gene mutations in AC patients in our cohort than in the TCGA cohort. The *BRIP1* and *WRN* genes are important genes for homologous recombination repair and targets for PARP inhibitors. Thus, cervical cancers with *BRIP1* and *WRN* alterations may be affected by PARP inhibitors.

The mutation frequencies of the *KRAS, TP53* and *ERBB2* genes in AC in our group were consistent with those in the TCGA cohort. Retrospective studies identified *ERBB2* gene mutations in 35–6% of cervical cancers and reported that *ERBB2* gene mutations may be associated with a poor prognosis [24, 31, 32]. Although these results need to be further analysed in larger sample sizes, our findings indicate that SCC and AC exhibit different molecular characteristics, which suggests that identifying specific targeted treatment strategies, such as PI3K and MEK inhibitors, may improve the clinical outcomes of these patients.

We also examined TMB in the samples and performed statistical analysis to identify gene mutations that were related to increased TMB. Previous studies have shown that a high TMB value is associated with an improved therapeutic effect of immunotherapy across cancers, including cervical cancer [33]. The TMB value could be extrapolated to the burden measured by WES, and therefore, it may be related to the predicted benefit of immune checkpoint inhibitors [34]. However, the use of WES to quantify TMB is currently not feasible in clinical practice. Rizvi et al. [35] showed that the predicted value of TMB by NGS has a good correlation with that of WES. A study verified the accuracy and predictive value of TMB evaluated by the targeted gene panel we used in the current study [36]. In our study, we calculated TMB using an NGS-based gene panel test. We found that the TMB value was associated with the cervical cancer subtype: SCC had a significantly higher TMB than AC. Our findings indicate that the overall mutational landscapes have significant differences between cervical cancer subtypes. Thus, the differences in the responses to immune checkpoint inhibitors of Chinese patients with different cervical cancer subtypes could be caused by the variance in gene alterations, PD-L1 expression and TMB status across different subtypes. Further studies using a large sample size are needed to test this hypothesis.

Interestingly, we also found that genetic mutations in *KMT2C* and *LRP1B* are independent predictors of TMB-H. To our knowledge, this is the first finding that genetic mutations in *KMT2C* and *LRP1B* are independent predictors of TMB-H in cervical cancer. *KMT2C,* a member of the KMT2 family, has been identified as a tumour suppressor that is frequently altered in multiple cancers [37]. Non-small-cell lung cancer patients with a mutated *KMT2C* gene have a higher TMB as well as PD-L1+/TMB-H, which are associated with a significantly longer median progression-free survival than those with wild-type *KMT2C* [38]. In our cohort, the *KMT2C* gene was the third most frequent gene mutation in cervical cancer and was also related to a higher TMB. These findings suggest the possibility of *KMT2C* gene mutation as a predictor of prognosis and response to immunotherapy and targeted therapy in cervical cancer. *LRP1B*, an endocytic low-density lipoprotein family receptor, is considered to be a candidate tumour suppressor that binds to extracellular ligands. Alterations in *LRP1B* were associated with high TMB in melanoma and non-small-cell lung cancer [39]. Although several studies have investigated the relationship between *LRP1B* and TMB, no studies have provided definitive findings for cervical cancer. Here, we found that mutations in *LRP1B* were associated with a higher TMB. *LRP1B*, as a result, may be defined as a predictive marker for the efficiency of CC immunotherapy.

The PTEN tumour suppressor is the second most commonly inactivated gene across cancer types. In a large cohort of non-small-cell lung cancer (NSCLC) patients, PTEN loss was associated with poorer prognosis [40]. Loss of PTEN has been associated with resistance to BRAF inhibitors and decreased overall survival in melanoma [41, 42]. Similarly, the results of this study show that PTEN mutations are associated with worse clinical outcomes. However, the secondary analysis results of the CLAP trial suggest that PTEN alterations were associated with improved outcomes in patients with cervical cancer treated with PD-1 inhibitor combination therapy, conferred longer PFS (*p* = 0.05) and trended towards increased OS (*p* = 0.08) [7]. PTEN mutations are associated with worse prognosis, but patients with PTEN mutations may benefit from anti-PD-1 combination therapy.

The study had several limitations. One limitation of this paper is the insufficient sample size. Another limitation is the lack of direct data for assessing the relationship between gene mutations and clinical prognosis. The third limitation is the heterogeneity of patient baselines. The fourth limitation is the TMB cut-off value was derived from the pan-cancer tumor. The fifth limitation is the absence of HPV status. However, it is well known that 99.7% of cervical cancers are associated with HPV infection [43], and squamous cell carcinoma and adenocarcinoma are the major pathological types of cervical cancer. HPV-independent cervical cancer including gastric type adenocarcinoma, clear cell adenocarcinoma, mesonephric adenocarcinoma and endometrioid adenocarcinoma [44]. This study including patients with squamous cell carcinoma or adenocarcinoma, and the relationship between HPV status and prognosis may not be positive. Therefore, further research is needed to investigate the connection between genetic alterations and the efficiency of immunotherapy in Chinese patients with cervical cancer.

## Conclusions

In conclusion, our study revealed the genetic landscape of cervical cancer in a relatively large sample of Chinese patients. We also identified the different genomic landscapes and immune characteristics of cervical SCC and AC. These findings may provide a basis for discovering potential therapeutic targets and improving the treatment of cervical cancer patients. We also showed that cervical cancers with mutations in the *KMT2C* and *LRP1B* genes have an increased TMB, and our data indicate that *KMT2C* and *LRP1B* gene mutations may serve as biomarkers to forecast therapeutic reactions. More importantly, one critical gene, PTEN, was identified to function as a prognostic marker for cervical cancer patient survival, providing insights for the application of diagnostic and prognostic tools. Nevertheless, large-sample prospective studies are still needed to verify our conclusions.

## Supplementary Information

**Additional file 1: Table S1**. Gene mutations in the TCGA cohort.

**Additional file 2: Table S2**. Clinicopathological features of cervical cancer patients.

**Additional file 3: Figure S1**. Kaplan-Meier estimated **A** PFS and **B** OS by PD-L1 expression.

## Data Availability

Our cohort datasets generated and analysed during the current study are available in the NCBI SRA repository, under accession PRJNA851600 (https://www.ncbi.nlm.nih.gov/bioproject/PRJNA851600).

## Abbreviations

SCC: Squamous cell carcinoma
AC: Adenocarcinoma
TCGA: The Cancer Genome Atlas
NGS: Next-generation sequencing
TMB: Tumour mutational burden
CPS: Combined positive score
SNVs: Single nucleotide variants

## Footnotes

1 Qin Xu, et al. Genomic profiling and PD-L1 expression of advanced cervical cancer to predict response to programmed death-1 inhibitor combination therapy: a secondary analysis of the CIBI308ALTER-C201 trial [abstract]. Gynecologic Oncology (unpublished observations).

2 Qin Xu, et al. Genomic profiling and PD-L1 expression of advanced cervical cancer to predict response to programmed death-1 inhibitor combination therapy: a secondary analysis of the CIBI308ALTER-C201 trial [abstract]. Gynecologic Oncology (unpublished observations).
