# Supplementary material for: Genomic landscape, immune characteristics and prognostic mutation signature of cervical cancer in China
Source: BMC Med Genomics. 2022 Nov 4;15:231. doi: 10.1186/s12920-022-01376-9 (PMC9636686; doi:10.1186/s12920-022-01376-9)
Supplement: Supplementary file 2 — Additional file 2: Table S2. Clinicopathological features of cervical cancer patients. [file 12920_2022_1376_MOESM2_ESM.docx]

**Table S2.** Clinicopathological features of cervical cancer patients

| Characteristic | Value (%) |
| --- | --- |
| Age |  |
| Median | 50.5 |
| Range | 34-73 |
| Histological type |  |
| Squamous cell carcinoma | 67(68.4) |
| Adenocarcinoma | 26(26.5) |
| Adenosquamous carcinoma | 5(5.1) |
| FIGO |  |
| I | 16(16.3) |
| II | 34(34.7) |
| III | 31(31.6) |
| IV | 11(11.2) |
| Unknown | 6(6.1) |
| PD-L1 expression |  |
| Negative | 21(21.3) |
| Positive | 54(55.1) |
| Unknown | 23(23.5) |
| FIGO：Federation International of Gynecology and Obstetrics | |
