# Supplementary figures and images for: Genomic landscape, immune characteristics and prognostic mutation signature of cervical cancer in China
Source: BMC Med Genomics. 2022 Nov 4;15:231. doi: 10.1186/s12920-022-01376-9 (PMC9636686; doi:10.1186/s12920-022-01376-9)

**
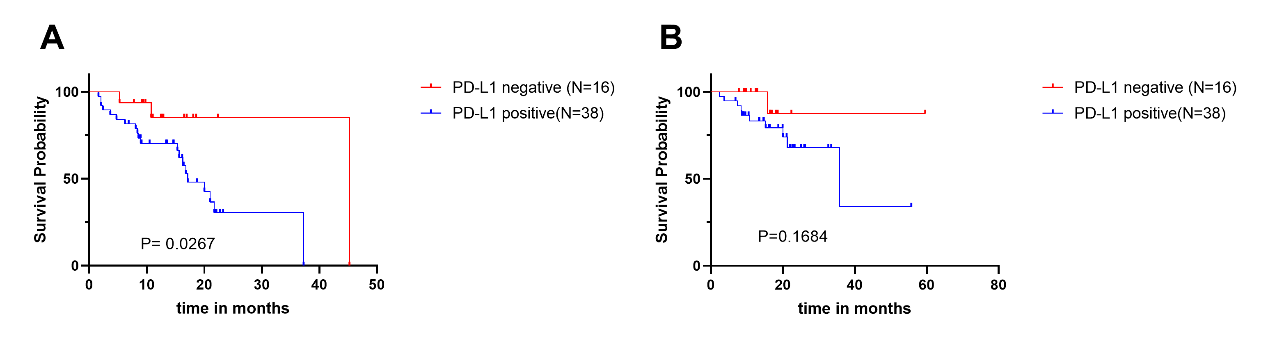
**

**Figure S1. Kaplan-Meier estimated A] PFS and B] OS by PD-L1 expression.**

Supplement: Supplementary file 3 — Additional file 3: Figure S1. Kaplan-Meier estimated A PFS and B OS by PD-L1 expression. [file 12920_2022_1376_MOESM3_ESM.docx]
